# Metabolic Syndrome in Non-diabetic Stroke Patients

**DOI:** 10.7759/cureus.72972

**Published:** 2024-11-04

**Authors:** Ahmed Wahab, Jamil Muqtadir, Abdul R Ansari, Muhammad Tahseen, Kashif Ayoob, Syed Haris M Zaidi, Arhama S Muhammad, Aisha Khan, Sehar Ahmed

**Affiliations:** 1 Internal Medicine, Ziauddin University, Karachi, PAK; 2 Infectious Diseases, Ziauddin University, Karachi, PAK; 3 Infectious Diseases, Dr. Ziauddin Hospital, Karachi, PAK; 4 Internal Medicine, Dr. Ziauddin Hospital, Karachi, PAK; 5 Medicine, Ziauddin University, Karachi, PAK

**Keywords:** elderly, ischemic stroke, metabolic syndrome, non-diabetic, vascular damage

## Abstract

Introduction

Metabolic syndrome (MetS) encompasses a range of diverse conditions, such as hypertension, hyperglycemia, central obesity, dyslipidemia, and diabetes. MetS in non-diabetic elderly patients with acute ischemic stroke can worsen vascular damage and lead to worse outcomes, highlighting the significance of early detection.

Objective

The objective of this paper was to determine the frequency of MetS in non-diabetic elderly patients with acute ischemic stroke visiting a tertiary care hospital.

Material and methods

This study was carried out in the medical department of Dr. Ziauddin Hospital in Karachi, Pakistan for a duration of six months from June 20, 2023, to December 19, 2023, following the adoption of the synopsis. All patients meeting the specified criteria and attending Dr. Ziauddin Hospital in Karachi were enrolled in the research. Informed consent was obtained after a thorough description of the methods, possible dangers, and advantages of the study.

Each patient underwent the metabolic assessment according to the International Diabetes Federation (IDF) criteria. Data collected was recorded in the provided proforma and electronically utilized for research endeavors. The analysis was conducted utilizing SPSS version 26.0 (IBM Corp., Armonk, NY). Descriptive statistics were computed, and the chi-square/Fisher's exact test was utilized for stratified analysis, with a p-value < 0.05 deemed significant.

Result

The study included patients aged between 65 and 90 years, with a median age of 74. Among the total population assessed, 133 individuals were male (76.4%) and 41 were female (23.6%). MetS was identified in 116 patients, representing 66.7% of the study population.

Conclusion

It is to be concluded that MetS was highly prevalent in non-diabetic elderly patients with acute ischemic stroke. This highlights a significant relationship between MetS and the occurrence of cerebrovascular accidents in this demographic. Further exploration and potential interventions targeting MetS in this population could be beneficial for improving health outcomes.

## Introduction

One of the top four major causes of morbidity and death worldwide is stroke [1]. This is an acute vascular-origin neurologic damage. For epidemiological research, a common definition of stroke is essential [2]. The World Health Organization (WHO) defines a stroke as fast-progressing clinical evidence of a focal (or global) disruption of cerebral function lasting for 24 hours or more, or resulting in death and has no other evident cause than vascular origin [3]. Global statistics show that ischemic strokes with reduced blood flow (infarction) to the brain or spinal cord account for 71% of all strokes [4]. Over 9 million people worldwide have survived strokes, whereas an estimated 4.5 million people die from them each year [5].

With an estimated population of 220 million, Pakistan ranks as the sixth-largest country by population in the world [6]. Currently, 41% of Pakistan's overall disease burden is attributable to non-communicable disorders, such as stroke [7]. Approximately 4.8% of individuals in a densely populated nation, such as Pakistan, may experience a stroke [8]. Given Pakistan's projected population of 220 million in 2023, this prevalence equates to roughly 10.56 million individuals. The annual incidence of stroke in the United States is roughly 795,000 instances, within a population of around 332 million (2023 estimate). Numerous international studies have shown a reduction in the incidence and mortality of stroke in non-diabetic groups [9]. Due to a larger loss in disability-adjusted life years, an estimated 94% of stroke deaths in South Asia occur in those under 70, compared to only 6% in nations with developed economies [10]. Stroke, diabetes, and other prevalent illnesses frequently coexist. One significant independent risk factor for ischemic stroke was diabetes. The risk of an ischemic stroke was elevated by diabetes [11].

Metabolic syndrome (MetS) can be characterized as a substantial risk factor for vascular diseases, such as stroke [12]. Central obesity, higher triglycerides, diabetes, elevated blood pressure, and decreased high-density lipoprotein (HDL) are the main causes of MetS [13]. There have been reports linking MetS to age, sex, smoking, alcohol use, and physical activity [14]. Globally, concerns are growing about MetS as a public health issue. Worldwide reports of MetS prevalence range from 14% to 30% [15]. However, 66.96% of non-diabetic patients with acute stroke had MetS, according to another study [16]. It is postulated that the growth of CVD, which may result from dysregulation of fat metabolism, is significantly influenced by the inflammatory process in MetS [15, 16]. In a study by Hu on patients with MetS, 32.1% were categorized as obese and 63.1% had dyslipidemia [17]. In another study by Liang et al., 76.6% of patients with MetS had high blood pressure, 32.8% had high triglycerides, and 51.7% had low HDL-C [18]. Additionally, Naeem et al. found that elevated fasting blood glucose levels (diabetes) were observed in 39.9% of the study participants [19]. MetS is prevalent in non-diabetic elderly patients with acute stroke, highlighting the importance of considering the metabolic risk factors beyond individual components. The presence of MetS in this population has significant clinical implications for risk assessment and tailored interventions to reduce the risk of recurrent stroke and cardiovascular events [20, 21].

Rationale

The frequency of MetS in non-diabetic elderly patients with acute stroke deserves attention, as studies have shown that this condition is common in non-diabetic individuals and may pose a significant risk for cardiovascular diseases, including stroke [15-18].

Objective

To determine the frequency of MetS in non-diabetic elderly patients with acute ischemic stroke visiting a tertiary care hospital.

## Materials and methods

Study design

This was a cross-sectional study conducted at the Medicine Department of Dr. Ziauddin Hospital from June 20, 2023, to December 19, 2023, for a period of six months.

Sample size

Using the WHO sample size calculator, the frequency of MetS was taken as (66.96%) [16] in non-diabetic patients with acute stroke, the margin of error was 7%, and the confidence level was 95%. The estimated sample size was set at 174.

Inclusion criteria

The subjects included in this research were 65 to 90 years old, non-diabetic individuals of either gender, who presented with a history of acute ischemic stroke more than 24 hours after its onset.

Exclusion criteria

The individuals excluded from this study had preexisting conditions such as substance abuse, infection, metabolic disorders, or any other systemic disease. Patients who were assigned a modified Rankin Scale (mRS) scored higher than 2. Hemorrhagic stroke patients are identified based on the presence of hyperdensity on a brain CT scan. This study excluded patients with renal failure who had a serum creatinine level greater than 2 mg/dL, as well as patients with end-stage organ failure or a terminal malignancy.

Operational definition

To be diagnosed with MetS, a patient must have a BMI greater than 30 kg/m² or a waist circumference above the ethnic threshold, along with two of the following five components: a waist circumference of ≥ 102 cm (40 inches) in men or ≥88 cm (35 inches) in women; triglyceride levels ≥150 mg/dL (1.7 mmol/L); HDL cholesterol levels <40 mg/dL (1.03 mmol/L) in men or <50 mg/dL (1.29 mmol/L) in women; blood pressure of ≥130/85 mmHg; and a fasting glucose level of ≥110 mg/dL (5.6 mmol/L).

Data collection

Following acceptance of the synopsis by the College of Physicians and Surgeons Pakistan Research Department (CPSP), data collection was initiated. All patients who met the study's inclusion requirements and visited Dr. Ziauddin Hospital's outpatient or emergency room in Karachi were enrolled. Before being included in the study, the next of kin or attendees of each patient gave written informed consent. The researcher took both the examination and the full history. Under the guidance of a consultant with more than five years of expertise, the researcher evaluated each patient for metabolic conditions using the IDF criteria, as stated in the operational definition. The proforma (included as an annexure) was filled out with all of the clinical and demographic data.

Before study participants could be enrolled, strict adherence to the selection criteria was required to adjust for confounders and modifiers. Participants in the study were healthy adults, of both sexes, between the ages of 65 and 90, who had experienced an acute ischemic stroke more than 24 hours prior to its onset. Patients with any kind of systemic disease, infection, intoxication, or metabolic disorders were not included in our patient pool. Individuals with scores on the modified Rankin Scale (mRS) greater than 2 were excluded. Patients who had suffered a hemorrhagic stroke and were detected by a brain CT scan with hyper-density were also excluded. Patients with end-stage organ failure, terminal cancer, or renal failure (defined as a blood creatinine level higher than 2 mg/dL) were excluded from the study. By removing variables that could potentially skew the data or add bias to the research, these criteria guaranteed that the study's primary emphasis would be on older, non-diabetic patients who had acute ischemic strokes. A pre-made proforma was used to collect and document the whole data and it was kept confidential, and it remained accessible to authorized individuals only.

Data Analysis

The data was analyzed using SPSS version 26.0 (IBM Corp, Armonk, NY). The normality of the continuous data was tested using the Shapiro-Wilk test. Mean and SD / median with IQR as appropriate were calculated for age, weight, height, BMI, and duration of acute ischemic stroke. Frequencies and percentages were calculated for gender, residential status, and outcome variable i.e. MetS (yes/no). Data was stratified on the basis of age, gender, BMI, duration of acute ischemic stroke, and residential status to see the effect of these on outcome variables. Post-stratification, chi-square/Fisher’s exact test as appropriate was applied; a p-value ≤ 0.05 was deemed significant.

## Results

This study aimed to investigate the occurrence of MetS in non-diabetic elderly individuals with acute ischemic stroke who were receiving treatment at a tertiary care hospital. A total of 174 patients were included in the study. The complete descriptive statistics are shown in Table 1. The distribution of continuous variables was assessed using the Shapiro-Wilk test for age (P= 0.390), height (P=0.0001), weight (P=0.0001), body mass index (P= 0.069), and duration of acute ischemic stroke (P= 0.394), as presented in Table 2.

**Table 1:** Descriptive statistics The data has been represented as medians and IQRs IQR: interquartile range

Variable	Median (IQR)
Age (years)	74 (65-90)
Height (cm)	170 (155-182)
Weight (Kg)	77 (55-101)
Body Mass index (Kg/m2)	25.82 (19.03-39.26)
Duration of Acute Ischemic Stroke (hours)	42.0 (26.0-75.0)

**Table 2:** Descriptive statistics of Shapiro-Wilk test (n=174) The data is given as mean±SD SD: standard deviation *Fisher's exact test, ** Chi-square test

Variable	Mean ± SD	P-Value
Age	76.22±7.89	0.390*
Height	168.80±8.28	0.0001**
Weight	77.00±11.71	0.0001**
Body mass index	27.13±4.52	0.069*
Duration of acute ischemic stroke	43.49±10.94	0.394*

The patients' ages ranged from 65 to 90 years, with a median of 74 and an interquartile range of 16. The patients' weights ranged from 55 to 101 kg, with a median of 77 and an interquartile range of 15. The patients' heights ranged from 155 to 182 cm, with a median of 170 and an interquartile range of 15. The patients' body mass index ranged from 19.03 to 39.26 kg/m2, with a median of 25.82 and an interquartile range of 7. The duration of acute ischemic stroke in the patients ranged from 26 to 75 hours, with a median of 42 and an interquartile range of 13.3. These values are shown in Table 2.

The study included a total of 174 patients, with a demographic breakdown showing that 133 individuals (76.4%) were male and 41 individuals (23.6%) were female. Regarding, 85 participants (48.9%) lived in urban areas, while 89 participants (51.1%) resided in rural areas. Additionally, MetS was found in 116 patients, representing 66.7% of the study population.

**Figure 1:**
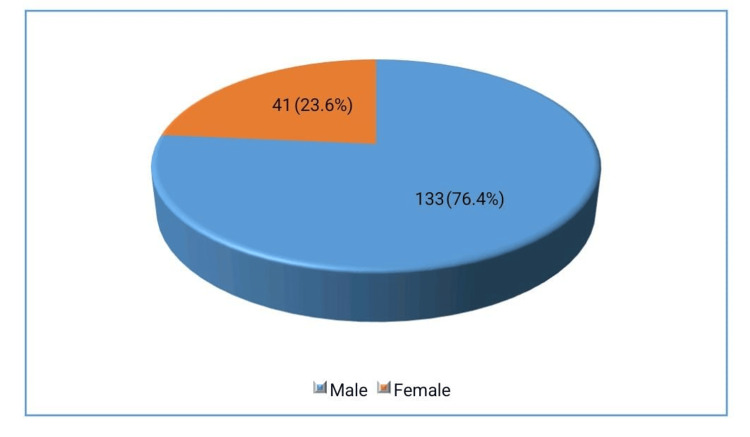
Frequency of gender (n=174)

**Figure 2:**
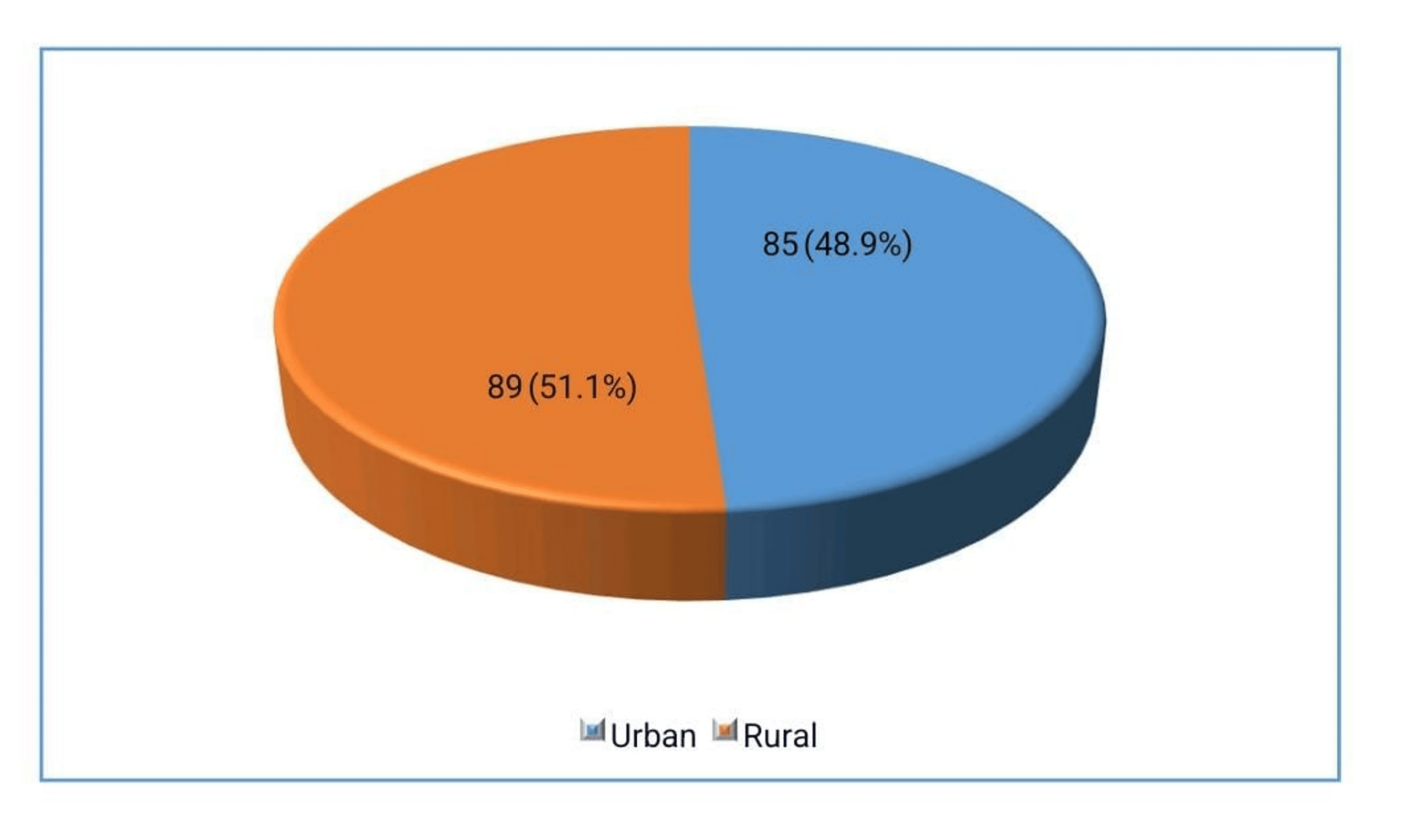
Frequency of residential status (n=174)

**Figure 3:**
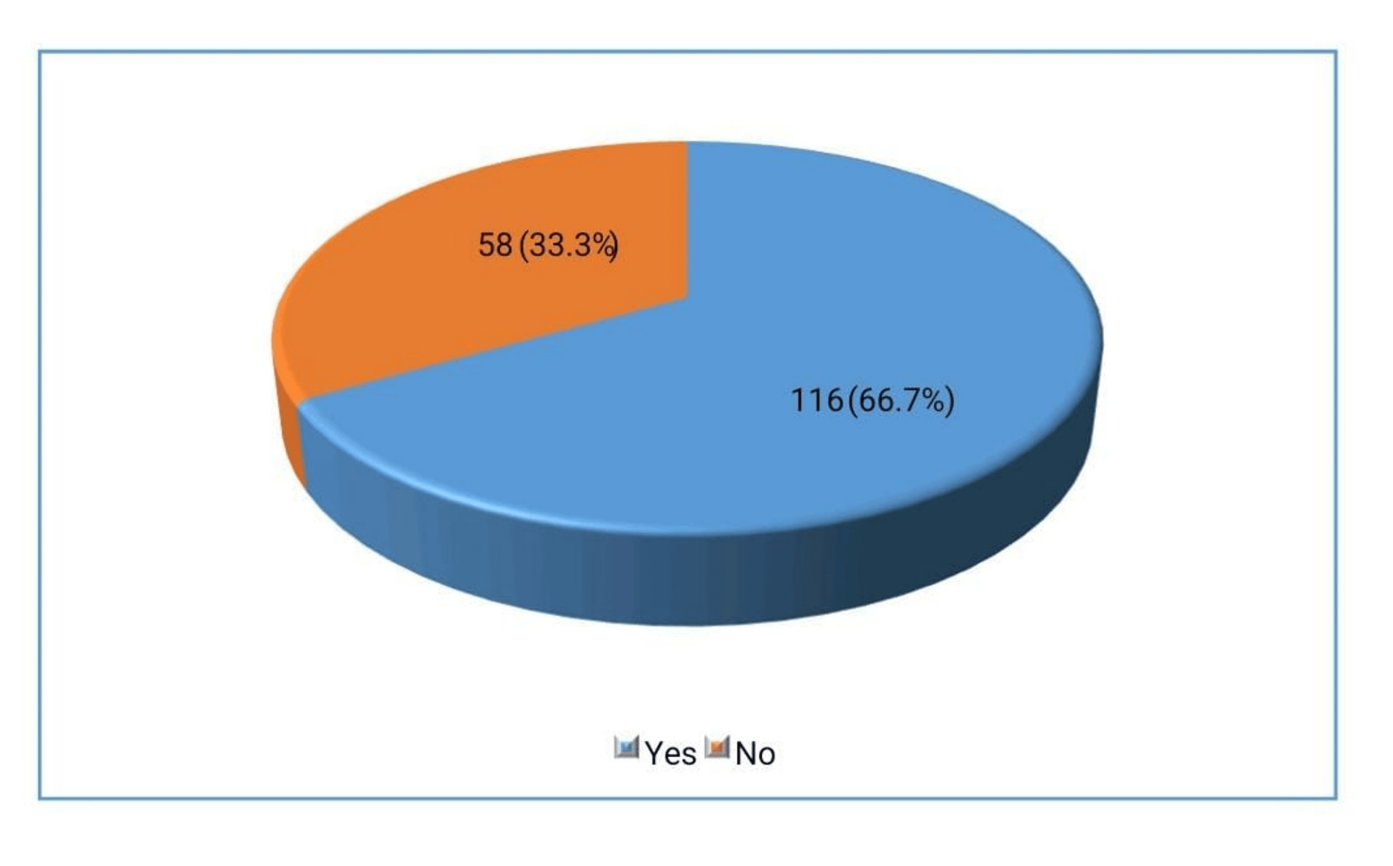
Frequency of MetS (n=174) MetS: metabolic syndrome

These distributions are visually represented in Figures 1-3 respectively.

In the age group of 65-75 years, 72 (41.4%) individuals had MetS while among those aged over 75, 44 (25.3%) individuals had MetS. The p-value associated with the comparison is 0.390. In the BMI range of 19-24 kg/m², 31 (17.8%) individuals had MetS, while among those with a BMI over 24, 85 (48.9%) had MetS. The associated p-value for the comparison is 0.069. In the 26-40-hours duration group, 56 (32.2%) participants had MetS compared to 30 (17.2%) without it. For durations exceeding 40 hours, 60 (34.5%) participants had MetS, while 28 (16.1%) did not. The p-value for comparison is 0.394. Among males, 90 (51.7%) participants hadMetS compared to females, 26 (14.9%) participants had MetS. The p-value for comparison is 0.372. In urban areas, 54 (31.0%) individuals were found to have MetS. Conversely, in rural regions, 62 (35.6%) individuals had MetS. The p-value associated with the comparison is 0.243 as shown in Table 3.

**Table 3:** Stratification of various groups The data has been represented as n and percentage (%) BMI: body mass index; MetS: metabolic syndrome *Fisher's exact test

Variables		MetS		P-value
		Yes	No	
Age (in years)	65-75	72 (41.4%)	34 (19.5%)	0.390*
	>75	44 (25.3%)	24 (13.8%)	
BMI (kg/m2)	19-24	31 (17.8%)	9 (5.2%)	0.069*
	>24	85 (48.9%)	49 (28.2%)	
Acute ischemic stroke (hours)	26-40	56 (32.2%)	30 (17.2%)	0.394*
	>40	60 (34.5%)	28 (16.1%)	
Residential status	Urban	54 (31.0%)	31 (17.8%)	0.243*
	Rural	62 (35.6%)	27 (15.5%)	
Gender	Female	26 (14.9%)	15 (8.6%)	0.372*
	Male	90 (51.7%)	43 (24.7%)	

## Discussion

MetS has emerged as a significant health concern globally, particularly among the elderly population, predisposing them to various cardiovascular ailments, including acute ischemic stroke (AIS) [3]. AIS represents a leading cause of morbidity and mortality among the elderly, necessitating a comprehensive understanding of associated risk factors to optimize management strategies [22]. MetS encompasses a range of diverse conditions, such as hypertension, hyperglycemia, central obesity, dyslipidemia, and diabetes. MetS in non-diabetic elderly patients with acute ischemic stroke can worsen vascular damage and lead to worse outcomes, highlighting the significance of early detection.

While MetS is often associated with diabetes mellitus, its impact extends beyond diabetic populations to encompass non-diabetic individuals, especially in the elderly demographic. Non-diabetic elderly patients with AIS and concomitant MetS present unique challenges due to the intricate interplay between aging, metabolic dysregulation, and cerebrovascular pathology [3]. Understanding the prevalence, clinical characteristics, and prognostic implications of MetS in this subset of patients is pivotal for tailored therapeutic interventions and prognostic stratification.

This study aimed to provide a foundational understanding of the intersection between MetS and AIS in non-diabetic elderly patients, highlighting the urgency of addressing metabolic derangements in acute cerebrovascular events. By elucidating the intricate relationship between MetS and AIS in this vulnerable population, healthcare providers can devise targeted strategies aimed at mitigating the burden of stroke and improving clinical outcomes. The discussion of MetS in non-diabetic elderly patients with acute ischemic stroke encompasses a multifaceted analysis of its prevalence, clinical implications, management challenges, and prognostic significance.

Firstly, the prevalence of MetS in non-diabetic elderly AIS patients underscores its substantial contribution to cerebrovascular pathology beyond diabetic populations [22]. Studies have reported a high prevalence of MetS in AIS patients, with rates ranging from 30% to 60% in non-diabetic individuals, highlighting the significance of metabolic dysregulation as a risk factor for stroke in the elderly demographic [23]. This prevalence underscores the necessity of routine metabolic screening in elderly stroke patients, irrespective of diabetic status, to identify and address underlying metabolic abnormalities.

Clinically, the presence of MetS in non-diabetic elderly AIS patients often exacerbates stroke severity and complicates management [24]. Metabolic abnormalities such as central obesity, dyslipidemia, and hypertension contribute to a pro-inflammatory and pro-thrombotic milieu, exacerbating cerebrovascular damage and impairing neurological recovery [25]. Moreover, insulin resistance, a hallmark of MetS, adversely impacts glucose metabolism and worsens stroke outcomes by exacerbating cerebral ischemia and promoting secondary brain injury.

The management of non-diabetic elderly AIS patients with concomitant MetS poses unique challenges due to the need for comprehensive risk factor modification and stroke prevention strategies [26]. While standard AIS management protocols prioritize thrombolytic therapy and secondary prevention measures, addressing metabolic abnormalities is equally crucial for mitigating recurrent stroke risk and improving long-term outcomes [27]. Lifestyle modifications, such as changes in diet, regular exercise, and maintaining a healthy weight, play a crucial role in reducing the risk of cardiovascular problems and lessening the impact of MetS in this group of people.

Furthermore, pharmacological interventions targeting individual MetS components, such as statins for dyslipidemia and antihypertensive agents for hypertension, may confer additional benefits in reducing stroke recurrence and improving vascular health. However, the optimal pharmacological approach must be individualized based on the patient's clinical profile, comorbidities, and risk-benefit considerations [28]. The prognostic implications of MetS in non-diabetic elderly AIS patients are significant, with several studies highlighting its association with increased mortality, recurrent stroke, and long-term disability [29, 30]. Metabolic abnormalities exacerbate stroke severity, hinder functional recovery, and predispose patients to vascular events, underscoring the prognostic significance of addressing MetS components in the acute and post-acute phases of stroke management [30].

This study found MetS in 116 (66.7%) patients with stroke. In another study, the prevalence of MetS has been reported as 14-30% globally [15], while another study reported the prevalence of MetS as 66.96% in non-diabetic patients with acute stroke [16].

This study elucidates the multifaceted impact of MetS on non-diabetic elderly AIS patients, emphasizing the need for holistic stroke management strategies that address both cerebrovascular and metabolic abnormalities. By recognizing the clinical implications of MetS in this vulnerable population, healthcare providers can optimize stroke care delivery and improve outcomes through targeted risk factor modification and personalized interventions tailored to individual patient needs.

This research is subject to several limitations, including sample selection bias, a cross-sectional study design, limited generalizability, and the presence of confounding factors like comorbidities and socioeconomic status. The study was conducted at a solitary tertiary care facility, which may not be representative of the total population. Due to the cross-sectional nature of the study methodology, it is challenging to prove a causal relationship, and the findings may not be applicable to populations other than the specific one analyzed.

## Conclusions

It is to be concluded that MetS was highly prevalent in non-diabetic elderly patients with acute ischemic stroke. This highlights a significant relationship between MetS and the occurrence of stroke in this demographic. Further exploration and potential interventions targeting this condition in this population could be beneficial for improving health outcomes.
